# PD28 - Is other bovid mammals milk tolerated by children who have been submitted to cow’s milk oral immunotherapy?

**DOI:** 10.1186/2045-7022-4-S1-P28

**Published:** 2014-02-28

**Authors:** Mònica Piquer Gibert, Spera Adriana Machinena, Montserrat Alvaro Lozano, Maria Teresa Giner Muñoz, Olga Domínguez Sánchez, Jaime Lozano Blasco, Ana Maria Plaza Martin

**Affiliations:** 1 Hospital Sant Joan de Déu, Barcelona, Spain

## Introduction

Caseins cross-reactivity rises up to 90% between cow’s milk (CM) and other bovid milks. There are few studies on tolerance to other mammals milk in patients submitted to cow’s milk oral immunotherapy (CMOIT). AIMS: To determine the percentage of patients whom after CMOIT tolerate water buffalo (WB), goat and sheep’s milk proteins (MP). To describe immunologic features which can predict allergy.

## Methods

Inclusion criteria: children submitted to CMOIT tolerating >150cc CM daily, more than 1 year in maintenance phase. We evaluated: total IgE, skin prick test (SPT) to CMP, WB, goat and sheep’s MP; specific IgE (sIgE) and IgG4 to CMP, goat and sheep’s MP. Patients underwent oral food challenge (OFC) to WB, goat and sheep’s MP.

## Results

51 children, 28 males, mean age at CMOIT 6.6 years (2.2-14.2) and 9.2 at OFC with WB, goat and sheep’s MP. At OFC to WB, goat and sheep’s MP: 47, 30 and 32 patients tolerated; 3, 7 and 4 non-anaphylactic reaction; 1, 12 and 11 anaphylaxis; 0, 2 and 4 didn’t undergo OFC, respectively. Significant differences between tolerant and non-tolerant in SPT to casein, goat and sheep’s MP, and sIgE to CM, casein, goat and sheep’s MP were observed. The area under the ROC curve for goat’s sIgE relating tolerance was 0.92, p<0.05, cut-off point 5.66 KUI/L (89.5% sensibility-96.3% specificity); for sheep’s sIgE 0.87, p<0.05, cut-off point 7.69 KUI/L (80% sensibility-86.7% specificity). CAP inhibition assays (solid phase goat and sheep, tolerant vs non-tolerant sera pools, CM as inhibitor) without significant differences although in the tolerant pool a greater inhibition is observed.

## Conclusions

58.8% of the patients who have undergone CMOIT tolerate other bovid MP. 92.15% tolerate WBMP. In OFC to goat and sheep’s MP 23.95% have experienced anaphylaxis and only 1.96% in WBMP OFC. Our cut-off points can predict adverse events.

